# Mechanisms Involved in the Beneficial Effects of Spironolactone after Myocardial Infarction

**DOI:** 10.1371/journal.pone.0076866

**Published:** 2013-09-30

**Authors:** Marcos F. Minicucci, Priscila P. dos Santos, Bruna P. M. Rafacho, Andrea F. Gonçalves, Renata A. C. Silva, Fernanda Chiuso-Minicucci, Paula S. Azevedo, Bertha F. Polegato, Katashi Okoshi, Elenize J. Pereira, Sergio A. R. Paiva, Leonardo A. M. Zornoff

**Affiliations:** 1 Internal Medicine Department, Botucatu Medical School, University Estadual Paulista, Botucatu, São Paulo, Brazil; 2 Department of Microbiology and Immunology, Institute of Biosciences, University Estadual, Paulista, Botucatu, São Paulo, Brazil; Osaka University Graduate School of Medicine, Japan

## Abstract

**Introduction:**

Our objective was to analyze the effect of spironolactone on cardiac remodeling after experimental myocardial infarction (MI), assessed by matricellular proteins levels, cardiac collagen amount and distribution, myocardial tissue metalloproteinase inhibitor-1(TIMP-1) concentration, myocyte hypertrophy, left ventricular architecture, and *in* *vitro* and *in* *vivo* cardiac function.

**Methods:**

Wistar rats were assigned to 4 groups: control group, in which animals were submitted to simulated surgery (SHAM group; n=9); group that received spironolactone and in which animals were submitted to simulated surgery (SHAM-S group, n=9); myocardial infarction group, in which animals were submitted to coronary artery ligation (MI group, n=15); and myocardial infarction group with spironolactone supplementation (MI-S group, n=15). The rats were observed for 3 months.

**Results:**

The MI group had higher values of left cardiac chambers and mass index and lower relative wall thicknesses compared with the SHAM group. In addition, diastolic and systolic functions were worse in the MI groups. However, spironolactone did not influence any of these variables. The MI-S group had a lower myocardial hydroxyproline concentration and myocyte cross-sectional area compared with the MI group. Myocardial periostin and collagen type III were lower in the MI-S group compared with the MI-group. In addition, TIMP-1 concentration in myocardium was higher in the MI-S group compared with the MI group.

**Conclusions:**

The predominant consequence of spironolactone supplementation after MI is related to reductions in collagens, with discrete attenuation of other remodeling variables. Importantly, this effect may be modulated by periostin and TIMP-1 levels.

## Introduction

Heart failure is a frequent complication of myocardial infarction (MI). Several factors influence the appearance of left ventricular dysfunction after MI. However, cardiac remodeling is a major cause of progressive heart failure following coronary occlusion. Importantly, the consequences of cardiac dysfunction after MI are well established, and cardiac dysfunction increases the risk of death by at least 3-fold. It is well accepted that patients who have heart failure and left ventricular systolic dysfunction are at higher risk for adverse outcomes, including cardiac rupture, stroke, ventricular arrhythmias, recurrent myocardial infarction, and death, including sudden death [1].

Recent large clinical trials suggest that aldosterone receptor blockade improves survival and reduces morbidity in patients with heart failure and reduced ejection fraction [2-4]. However, to date, there is a poor understanding of the mechanisms involved in the beneficial effects of aldosterone receptor blockade in this scenario. Therefore, the objective of this study was to analyze the effect of spironolactone on cardiac remodeling after experimental MI; the effect was assessed by matricellular proteins, cardiac collagen amount and distribution, myocardial tissue metalloproteinase inhibitor-1 concentration, myocyte hypertrophy, left ventricular architecture, hemodynamic recording, and *in vitro* and *in vivo* cardiac function.

## Materials and Methods

All of the experiments and procedures were performed in accordance with the National Institute of Health’s Guide for the Care and Use of Laboratory Animals and were approved by the Animal Ethics Committee of Botucatu Medical School. All efforts were made to minimize suffering.

Male, Wistar rats that weighed 200-230 g were assigned to 4 experimental groups: a control group, in which animals were submitted to simulated surgery (SHAM group; n=9); a group in which animals received spironolactone (20 mg/kg of diet/day) and were submitted to simulated surgery (SHAM-S group, n=9); a myocardial infarction group, in which animals were submitted to coronary artery ligation (MI group, n=15); and a myocardial infarction group with spironolactone supplementation (MI-S group, n=15). An echocardiographic exam was performed 5 days after myocardial infarction, and there was no morphological or functional difference between the MI groups (data not shown). Water was supplied *ad libitum*. The rats were observed for 3 months, after which morphological, functional and biochemical analyses were performed.

### Coronary artery ligation

When the animals achieved body weights of 200-250 g, myocardial infarction was induced as previously described [5,6]. Briefly, the rats were anesthetized with ketamine (70 mg/kg) and xylazine (1 mg/kg), and after a left thoracotomy, the heart was exteriorized. The left atrium was retracted to facilitate the ligation of the left coronary artery with 5-0 mononylon between the pulmonary outflow tract and the left atrium. The heart was then replaced in the thorax, and the lungs were inflated by positive pressure as the thoracotomy was closed. The rats were housed in a temperature-controlled room (24°C) with a 12-hour light:dark cycle.

### Systolic blood pressure

The systolic pressure in the tails of the animals was measured using a tail plethysmograph with a polygraph (Byo-Sistem PE 300, NARCO), a sensor placed in the proximal region of the tail, and an electrosphygmomanometer to enable the recording of tail pressure. The animals were warmed in a wooden box at 37°C for 4 minutes, with the heat generated by 2 incandescent lamps, and were transferred to an iron support, where the tail was exposed. In the proximal region of the tail, a sensor (KSM-microphone) was placed and coupled to the plethysmograph. Blood pressure was recorded on paper with the polygraph at a velocity of 2.5 mm/s.

### Echocardiographic analysis

After 3 months, all of the animals were weighed and evaluated by a transthoracic echocardiographic exam [7,8]. All of the measurements were made by the same observer, according to the leading-edge method recommended by the American Society of Echocardiography/ European Association of Echocardiography [9]. The end-systolic and end-diastolic cavity areas were calculated as the sum of the areas from both the short- and long-axis views in diastole (SumD) and systole (SumS), respectively. The fractional area change (FAC) was calculated from the composite cavity areas as follows: FAC = (SumD-SumS)/SumD. Additionally, the left ventricular mass index (LVMI) was calculated using the following equation: LVMI = {[(LVEDD+2* LVWT)^3^ - (LVEDD)^3^]*1.04}/BW. The relative wall thickness (RWT) was determined as 2*posterior wall thickness/end-diastolic diameter. The velocities of transmitral diastolic flow (E and A velocities) were obtained from the apical four-chamber view. The E/A ratio, the isovolumetric relaxation time and the isovolumetric relaxation time corrected by the heart rate (IRT/RR^°5^) were used as indices of LV diastolic function.

### In vitro left ventricular function analysis

One day after the echocardiographic study, the rats were anesthetized with thiopental sodium (50 mg/kg i.p.) and administered heparin (2000 UI, i.p.). The chest was submitted to a median sternotomy under artificial ventilation. The entire heart was rapidly removed from the chest and transferred to a perfusion apparatus (model 830 Hugo Sachs Eletronick-Greenstasse). The ascending aorta was isolated and cannulated for retrograde perfusion with filtered and oxygenated Krebs-Henseleit solution, which was maintained at a constant temperature and perfusion pressure (37°C and 75 mmHg, respectively). All of the hearts were paced at 200 to 250 beats/min. The procedures and measurements were performed following a previously described method [10].

### Morphometric analysis

Upon completion of the functional analyses, the right and left ventricles (including the interventricular septum) were dissected, separated and weighed. Transverse sections of the LV were fixed in 10% buffered formalin and paraffin-embedded. Five-micron-thick sections were stained with hematoxylin and eosin (HE). The myocyte cross-sectional area was determined for a minimum of 100 myocytes per H&E-stained cross section. The measurements were obtained from digital images (400 × magnification) that were collected with a video camera attached to a Leica microscope; the images were analyzed with the Image-Pro Plus 3.0 software program (Media Cybernetics, Silver Spring, MD). The myocyte cross-sectional area was measured with a digital pad, and the selected cells were transversely cut so that the nucleus was in the center of the myocyte [11]. The lengths of the infarcted and viable muscle for both the endocardial and epicardial circumferences were determined by planimetry. Infarct size was calculated by dividing the endocardial and epicardial circumferences of the infarcted area by the total epicardial and endocardial ventricular circumferences. The measurements were performed on midventricular sections (5-6 mm from the apex) under the assumption that the left midventricular slice shows a close linear relation with the sum of the area measurements from all of the heart sections [12].

### Myocardial hydroxyproline concentration

The myocardial hydroxyproline concentration was utilized for fibrosis estimation. Hydroxyproline (HOP) was measured in the tissue (septum of LV and mid ventricular slice of RV) according to the method described previously [13]. Briefly, the tissue was dried for 4 h using a Speedvac Concentrator SC 100 that was attached to a refrigerated condensation trap (RVT 100) and vacuum pump (VP 100, Savant Instruments, Inc., Farmingdale, NY). The dry weight of the tissue was determined, and the samples were hydrolyzed overnight at 110 °C with 6 N HCl (1 ml/10 mg dry tissue). A 50-μl aliquot of the hydrolyzate was transferred to an Eppendorf tube and dried in the Speedvac Concentrator. Deionized water (1 ml) was added, and the sample was transferred to a tube with a Teflon screw cap. Potassium borate buffer (1 ml, pH 8.7) was added to maintain constant pH, and the sample was oxidized with 0.3 ml of chloramine T solution at room temperature for precisely 20 minutes. The oxidative process was stopped by adding 1 mL of 3.6 mol/l sodium thiosulfate and mixed thoroughly for 10 s. The solution was saturated with 1.5 g of KCl. The tubes were capped and heated in boiling water for 20 min. After cooling to room temperature, the aqueous layer was extracted with 2.5 ml of toluene. Next, 1 ml of toluene extract was transferred to a 12 x 75-mm test tube. Next, 0.4 ml of Ehrlich’s reagent was added to allow for the color to develop for 30 min. Absorbencies were read at 565 nm against a blank reagent. Deionized water and 20 μg/ml HOP were used as the blank and standard, respectively [14].

### Western blot analysis

Left ventricular samples were extracted using Tris-Triton buffer (10 mM Tris (pH 7.4), 100 mM NaCl, 1 mM EDTA, 1 mM EGTA, 1% Triton X-100, 10% glycerol, 0.1% SDS, 0.5% deoxycholate, 1 nM EDTA, 1 mM EGTA and a mixture of protease inhibitors, 1 mM sodium orthovanadate, 1 mM sodium fluoride and 1% leupeptin, aprotinin, pepstatin) to detect collagen III and I and periostin. The samples were then centrifuged at 12000 at 4 °C for 20 min, and the supernatant was collected. The supernatant protein content was quantified using the Bradford method. The samples were separated on a 10% SDS-polyacrylamide gel, and the proteins were transferred to a nitrocellulose membrane. The membrane was blocked with 5% nonfat dry milk in Tris-buffered saline containing Tris 1 M (pH 8.0), NaCl 5 M and Tween 20 at room temperature for 2 hours. The membrane was then incubated with primary antibody anti-Collagen III, mouse monoclonal IgG1 (Abcam, Inc., Canada, ab 6310), Collagen I A1, rabbit polyclonal IgG (Santa Cruz Biotechnology, Inc., Europe, sc 8784R), periostin and goat polyclonal IgG (Santa Cruz Biotechnology, Inc., Europe, sc 49480). The membrane was washed with TBS and Tween 20 and incubated with secondary peroxidase-conjugated antibody. A Super Signal® West Pico Chemiluminescent Substrate (Pierce Protein Research Products, Rockford, USA) was used to detect bound antibodies. GAPDH (GAPDH (6C5), mouse monoclonal IgG1, (Santa Cruz Biotechnology, Inc., Europe, sc 32233) was used for collagen III and I and periostin blot normalization.

### Metalloproteinase-2 and -9 activity

The metalloproteinase (MMP)-2 and -9 activity was determined as previously reported [10]. In brief, samples for analysis were prepared by dilution in extraction sample buffer consisting of 50mM Tris, pH 7.4 ; 0.2 M NaCl; 0.1% Triton X and 10 mM CaCl_2_. Then they were diluted in application sample buffer consisting of 0.5 M Tris, pH 6.8; 100% glycerol, and 0.05% bromophenol blue. The samples were loaded into the wells of 8% SDS-polyacrylamide containing 1% gelatin. Electrophoresis carried out in a Bio-Rad apparatus at 80V for 2 hours, when bromophenol blue reaches the bottom of the gel. The gel was removed and washed 2 times with 2.5% Triton-X-100 and then washed with 50mM Tris pH 8.4. The gel was then incubated at 37°C overnight in activation solution consisting of 50 mM Tris pH 8.4; 5 mM CaCl_2_ and Zn Cl_2_. The staining was performed for 2 hours with 0.5% comassie blue and destaining in 30% methanol and 10% acetic acid until clear bands over a dark background were observed. Staining and destaining were performed at room temperature on a rotatory shaker. The gels were photographed and the intensity of gelatinolytic action (clear bands) analyzed in UVP,UV, White Darkhon image analyzer.

### The evaluation of myocardial tissue metalloproteinase inhibitor-*1* concentration

The levels of TIMP-1 in the heart homogenates were evaluated by ELISA according to the manufacturer’s instructions (R & D Systems, Minneapolis, MN, USA).

### Statistical analysis

The data are expressed as the means ± SD. Comparisons between groups were performed by two-way ANOVA analysis followed by Holm-Sidak. For infarct size comparison, the Student’s t-test was performed. The data analysis was carried out with SigmaStat for Windows v2.03 (SPSS Inc., Chicago, IL). The significance level was set at P < 0.05.

## Results

There was no difference in infarct size between the MI and MI-S groups (MI: 33.17 ± 13.39% *vs.* MI-S: 25.06 ± 13.64%; p=0.174).

The echocardiographic data are listed in Table 1. The animals in the MI group had higher values for left cardiac chambers corrected by body weight, higher LVMI and lower relative wall thicknesses compared with the SHAM group. In addition, diastolic and systolic functions were worse in the MI groups in echocardiographic and *in vitro* analysis. However, spironolactone did not influence any of these variables (Tables 1 and 2). There were no differences in systolic blood pressure between the groups.

**Table 1:** Echocardiographic data.

	SHAM (n= 9)	SHAM-S (n= 9)	MI (n= 15)	MI -S (n= 15)	p MI	p S	p MIxS
BW (g)	486.6 ± 30.3	486.1 ± 31.7	458.5 ± 30.2	482.3 ± 55.2	0.190	0.336	0.318
HR (bpm)	298 ± 17	307 ± 30	311 ± 34	305 ± 29	0.536	0.811	0.402
LVDD/BW (mm/kg)	16.9 ± 0.9	16.3 ± 1.5	23.0 ± 2.5	21.8 ± 2.9	<0.001	0.170	0.717
LVSD/BW (mm/kg)*	7.88 ± 0.9	6.7 ± 0.9	17.4 ± 2.7	16.1 ± 3.4	< 0.001	0.019	0.480
RWT	0.34 ± 0.02	0.36 ± 0.02	0.29 ± 0.03	0.30 ± 0.03	<0.001	0.162	0.369
LVMI (g/kg)*	1.72 ± 0.20	1.59 ± 0.14	3.29 ± 0.96	2.90 ± 0.58	<0.001	0.151	0.799
IRT/RR^0.5^ (ms)	48.0 ± 4.8	52.3 ± 6.5	51.5 ± 12.9	59.8 ± 11.1	0.077	0.044	0.502
E/A*	1.43 ± 0.17	1.38 ± 0.24	3.92 ± 3.87	2.47 ± 2.21	0.141	0.507	0.839
EDT (ms)	38.6 ± 6.33	43.7 ± 7.52	34.8 ± 6.07	37.4 ± 6.88	0.022	0.076	0.533
FAC	73.99 ± 7.85	69.84 ± 4.45	39.00 ± 11.05	34.62 ± 9.37	<0.001	0.120	0.966
PWSV (mm/s)	36.1 ± 3.92	33.7 ± 1.90	28.1 ± 3.92	28.6 ± 4.85	<0.001	0.431	0.238

S: spironolactone; MI: myocardial infarction; BW: body weight; HR: heart rate; LVDD: LV end-diastolic dimension; LVSD: LV end-systolic dimension; RWT: relative wall thickness; LVMI: left ventricle mass index; IVRT/RR^0.5^: isovolumetric relaxation time corrected for heart rate; E/A: peak velocity of early ventricular filling/ peak velocity of transmitral flow during atrial contraction; EDT: E wave deceleration time; FAC: fractional area change; PWSV: posterior wall shortening velocity. * data normalized for statistical analysis.

**Table 2:** In vitro left ventricular function and systolic blood pressure.

	SHAM (n= 6)	SHAM-S (n= 4)	MI (n= 6)	MI -S (n= 5)	p MI	p S	p MIxS
+ dp/dt max (mmHg/s)	4041 ± 1111	4594 ± 449	2437 ± 931	2150 ± 736	<0.001	0.739	0.298
- dp/dt max (mmHg/s)	2083 ± 385	2344 ± 359	1354 ± 490	1275 ± 311	<0.001	0.615	0.350
Systolic blood pressure (mmHg)#	130 ± 21.2	115 ± 20.3	119 ± 15.8	126 ± 17.9	0.898	0.400	0.052

S: spironolactone; MI: myocardial infarction; + dp/dt max: maximum rate of ventricular pressure rise; - dp/dt max: decreased maximum rate of ventricular pressure rise. # (SHAM=9; SHAM-S=9; MI=15; MI-S=15).

The morphological data are listed in Table 3. The BW-corrected left (LVW/BW) and right ventricular weights (RVW/BW) were elevated in the MI groups. No effect of spironolactone was observed. However, the MI-S group had a lower myocardial hydroxyproline concentration (MHOP) and myocyte cross-sectional area (CSA) compared with the MI group.

**Table 3:** Morphological data.

	SHAM (n= 9)	SHAM-S (n= 9)	MI (n= 16)	MI -S (n= 15)	p MI	p S	p MIxS
LVW/BW (g/mg)*	1.81 ± 0.21	1.54 ± 0.40	2.04 ± 0.54^•,ƒ^	1.88 ± 0.51	0.038	0.115	0.695
RVW/BW (g/mg)*	0.41 ± 0.11	0.34 ± 0.11	0.69 ± 0.33	0.52 ± 0.20	0.001	0.084	0.464
MHPO (mg/g)#	2.95 ± 0.21	3.42 ± 0.45	5.67 ± 0.70^•,ƒ^	4.17 ± 0.83	<0.001	0.121	0.008
CSA (µm^2^)**	315 ± 56	311 ± 67	483 ± 63^•,ƒ^	370 ± 56	<0.001	0.016	0.025

S: spironolactone; MI: myocardial infarction; BW: body weight; LVW: left ventricular weight; RVW: right ventricular weight; MHPO: myocardial hydroxyproline concentration; CSA: cross-sectional area. * data normalized for statistical analysis.

# (SHAM=4; SHAM-S=5; MI=4; MI-S=4.)

** (SHAM=8; SHAM-S=7; MI=6; MI-S=8.)

When interactions were observed, symbols show the significant differences (^•^ MI≠MI-S and ^ƒ^ MI≠SHAM)

Myocardial periostin and collagen type III were lower in the MI-S group compared with the MI-group. In addition, tissue metalloproteinase inhibitor-1 concentration in myocardium was higher in the MI-S group compared with the MI group (Table 4). On the other hand, in our study, MMP-2 and -9 levels were not affected by spironolactone treatment (Table 4).

**Table 4:** Myocardial collagen, periostin and tissue metalloproteinase inhibitor-1 concentrations.

	SHAM (n= 4)	SHAM-S (n= 5)	MI (n= 6)	MI -S (n= 6)	p MI	p S	PMIxS
Periostin/GAPDH*	0.001 ± 0.0006	0.002 ± 0.001^ϕ^	0.165 ± 0.113^•,ƒ^	0.045 ± 0.023	<0.001	0.360	0.002
Type 1 collagen/GAPDH	1.366 ± 0.182	2.918 ± 1.416	4.192 ± 1.183^ƒ^	3.001 ± 1.028	0.014	0.738	0.019
Type 3 collagen/GAPDH*	1.003 ± 0.183	1.469 ± 0.937	2.618 ± 1.152^•,ƒ^	1.568 ± 0.391	0.005	0.548	0.045
TIMP-1 (pg/g of protein)*	21.2 ± 4.3	32.9 ± 14.4	32.5 ± 14.1	150.7 ± 136.7	0.010	0.008	0.149
MMP-9 active/total	0.48 ± 0.35	0.85 ± 0.10	0.78 ± 0.23	0.84 ± 0.1	0.200	0.062	0.179
MMP-2 active/total	0.75 ± 0.1	0.68 ± 0.1	0.57 ± 0.1	0.57 ± 0.1	0.001	0.426	0.422

S: spironolactone; MI: myocardial infarction; MMP: matrix metalloproteinase; TIMP-1: tissue metalloproteinase inhibitor-1. * data normalized for statistical analysis.

When interactions were observed, symbols show the significant differences (^•^ MI≠MI-S; ^ƒ^ MI≠SHAM and ^ϕ^ SHAM-S≠MI-S)

## Discussion

The objective of this study was to analyze the mechanisms involved in the beneficial effects of spironolactone following myocardial infarction. Our data showed that spironolactone reduced LV myocardium hypertrophy, but without alterations in blood pressure, ventricular enlargement, cardiac geometry, or diastolic and systolic ventricular function. Spironolactone did reduce collagen types I and III, which are associated with alterations in periostin and TIMP-1 levels.

Although the concept that aldosterone receptor blockade improves survival and reduces morbidity in patients with heart failure and reduces ejection fraction is universally accepted, understanding the mechanisms involved in this phenomenon remains a challenge. However, it is well accepted that LV remodeling after MI is associated with increased risk for ventricular arrhythmias and sudden cardiac death. In the EPHESUS trial, the reduction in cardiovascular death induced by eplerenone was predominantly driven by decreased sudden cardiac death [15]. A recent meta-analysis found the same result [16]. Therefore, attenuation of cardiac remodeling by aldosterone receptor blockade might be associated with decreased malign arrhythmias. Importantly, there is a strong association between cardiac fibrosis and sudden cardiac death [17].

It is important to note that the effects of aldosterone receptor blockade on cardiac remodeling have been studied previously. Regardless of the complexity of the remodeling process, the term is frequently used as a synonym for ventricular enlargement associated with functional alterations after MI [18-21]. Indeed, in experimental models, some, but not all studies showed attenuation of left ventricular dimensions with aldosterone receptor blockade [22-24]. The same fact was observed in clinical trials [25-27]. Therefore, despite hard evidence of the attenuation of collagen levels, the effects of aldosterone receptor blockade on cardiac geometry and function are conflicting.

In the present study, spironolactone attenuated only myocyte hypertrophy but did not change blood pressure, ventricular enlargement, cardiac geometry or diastolic and systolic ventricular function. Therefore, our data suggest that these variables are most likely not involved in the beneficial effects of spironolactone on cardiac dysfunction after MI.

Another important issue is that the cardiac extracellular matrix is critical for the remodeling process and is composed of structural elements, such as collagen and other proteins, including fibronectin, proteoglycans and matricellular proteins. An important component of this extracellular matrix is a family of extracellular matrix-degrading enzymes, the metalloproteinases (MMPs). The MMPs are a family of more than 25 species of zinc-dependent proteases that play a pivotal role in collagen degradation. The activity of MMPs in the heart is controlled by the action of specific MMPs inhibitors or TIMPs, particularly TIMP-1. Importantly, it is well accepted that increased TIMP activity is associated with lower collagen degradation and subsequent fibrosis [28]. In the present study, the decrease in collagen levels induced by spironolactone was associated with higher levels of TIMP-1. On the other hand, in our study, MMP-2 and -9 levels were not affected by spironolactone treatment, suggesting that other MMP could be involved in this process. We therefore might conclude that the effects of spironolactone on cardiac fibrosis are, at least in part, mediated by TIMP-1 action, but not by MMP-2 and -9.

An additional mediator of the remodeling process is a family of structurally unrelated extracellular macromolecules referred to as matricellular proteins; these proteins play a limited role in tissue architecture but serve as links between cells and the matrix. Periostin is one of the most important matricellular proteins and plays a role in the maturation and differentiation of fibroblasts in the developing neonatal heart [29]. After acute MI, during the cardiac repair phase, periostin is released into the infarct and activates signaling pathways that are essential for the reparative process [30-33]. However, the role of periostin in chronic cardiac remodeling after MI is unknown. In the present study, MI induced a dramatic increase in periostin three months after coronary occlusion. Importantly, spironolactone decreased periostin levels. Therefore, considering the role of periostin as a modulator of collagen metabolism, we can infer that the decreased collagen content induced by spironolactone might be regulated by periostin.

In conclusion, we propose that the predominant consequence of aldosterone receptor blockade after MI is related to reductions in collagen, with discrete attenuation of other remodeling variables. Importantly, this effect might be modulated by periostin and TIMP-1 levels.

## References

[1] Minicucci MF, Azevedo PS, Polegato BF, Paiva SA, Zornoff LA (2011) Heart failure after myocardial infarction: clinical implications and treatment. Clin Cardiol 34: 410-414. doi:10.1002/clc.20922. PubMed: 21688276. 21688276 10.1002/clc.20922 PMC6652587

[2] Pitt B, Zannad F, Remme WJ, Cody R, Castaigne A et al. (1999) The effect of spironolactone on morbidity and mortality in patients with severe heart failure. Randomized Aldactone Evaluation Study Investigators. N Engl J Med 341: 709-717. doi:10.1056/NEJM199909023411001. PubMed: 10471456. 10471456 10.1056/NEJM199909023411001

[3] Pitt B, Remme W, Zannad F, Neaton J, Martinez F et al. (2003) Eplerenone, a selective aldosterone blocker, in patients with left ventricular dysfunction after myocardial infarction. N Engl J Med 348: 1309-1321. doi:10.1056/NEJMoa030207. PubMed: 12668699. 12668699 10.1056/NEJMoa030207

[4] Zannad F, McMurray JJ, Krum H, Van Veldhuisen DJ, Swedberg K et al. (2011) Eplerenone in patients with systolic heart failure and mild symptoms. N Engl J Med 364: 11-21. doi:10.1056/NEJMoa1009492. PubMed: 21073363. 21073363 10.1056/NEJMoa1009492

[5] Minicucci MF, Azevedo PS, Martinez PF, Lima AR, Bonomo C et al. (2011) Critical infarct size to induce ventricular remodeling, cardiac dysfunction and heart failure in rats. Int J Cardiol 151: 242-243. doi:10.1016/j.ijcard.2011.06.068. PubMed: 21719125. 21719125 10.1016/j.ijcard.2011.06.068

[6] Pfeffer JM, Finn PV, Zornoff LA, Pfeffer MA (2000) Endothelin-A receptor antagonism during acute myocardial infarction in rats. Cardiovasc Drugs Ther 14: 579-587. doi:10.1023/A:1007890126061. PubMed: 11300358. 11300358 10.1023/a:1007890126061

[7] Ardisson LP, Minicucci MF, Azevedo PS, Chiuso-Minicucci F, Matsubara BB et al. (2012) Influence of AIN-93 diet on mortality and cardiac remodeling after myocardial infarction in rats. Int J Cardiol 156: 265-269. 21095625 10.1016/j.ijcard.2010.10.128

[8] Paiva SAR, Novo R, Matsubara BB, Matsubara LS, Azevedo PS et al. (2005) β-carotene attenuates the paradoxical effect of tobacco smoke on the mortality of rats after experimental myocardial infarction. J Nutr 135: 2109-3113. PubMed: 16140884. 16140884 10.1093/jn/135.9.2109

[9] Lang RM, Bierig M, Devereux RB, Flachskampf FA, Foster E et al. (2005) Recommendations for chamber quantification: a report from the American Society of Echocardiography's guidelines and standards Committee and the chamber quantification writing group, developed in conjunction with the European Association of Echocardiography, a branch of the European Society of Cardiology. J Am Soc Echocardiogr 18: 1440-1463. doi:10.1016/j.echo.2005.10.005. PubMed: 16376782. 16376782 10.1016/j.echo.2005.10.005

[10] Azevedo PS, Minicucci MF, Chiuso-Minicucci F, Justulin LA Jr, Matsubara LS et al. (2010) Ventricular remodeling induced by tissue vitamin A deficiency in rats. Cell Physiol Biochem 26: 395-402. doi:10.1159/000320563. PubMed: 20798524. 20798524 10.1159/000320563

[11] Paiva SA, Zornoff LA, Okoshi MP, Okoshi K, Matsubara LS et al. (2003) Ventricular remodeling induced by retinoic acid supplementation in adult rats. Am J Physiol Heart Circ Physiol 284: H2242-H2246. PubMed: 12574000. 12574000 10.1152/ajpheart.00646.2002

[12] Zornoff LA, Paiva SA, Minicucci MF, Spadaro J (2009) Experimental myocardium infarction in rats: analysis of the model. Arq Bras Cardiol 93: 434-440. doi:10.1590/S0066-782X2009001000018. PubMed: 19936465. 19936465 10.1590/s0066-782x2009001000018

[13] Zornoff LAM, Matsubara BB, Matsubara LS, Paiva SAR, Spadaro J (2000) Early rather than delayed administration of lisinopril protects the heart after myocardial infarction in rats. Basic Res Cardiol 95: 208-214. doi:10.1007/s003950050183. PubMed: 10879622. 10879622 10.1007/s003950050183

[14] Matsubara LS, Matsubara BB, Okoshi MP, Cicogna AC, Janicki JS (2000) Alterations in myocardial collagen content affect rat papillary muscle function. Am J Physiol Heart Circ Physiol 279: H1534-H1539. PubMed: 11009438. 11009438 10.1152/ajpheart.2000.279.4.H1534

[15] Brandimarte F, Blair JEA, Manuchehry A, Fedele F, Gheorghiade M (2008) Aldosterone receptor blockade in patients with left ventricular systolic dysfunction following acute myocardial infarction. Cardiol Clin 26: 91-105. doi:10.1016/j.ccl.2007.08.011. PubMed: 18312909. 18312909 10.1016/j.ccl.2007.08.011

[16] Bapoje SR, Bahia A, Hokanson JE, Peterson PN, Heidenreich PA et al. (2013) Effects of Mineralocorticoid Receptor Antagonists on the Risk of Sudden Cardiac Death in Patients With Left Ventricular Systolic Dysfunction: A Meta-analysis of Randomized Controlled Trials. Circ Heart Fail 6: 166-173. doi:10.1161/CIRCHEARTFAILURE.112.000003. PubMed: 23403436. 23403436 10.1161/CIRCHEARTFAILURE.112.000003 PMC3893922

[17] Gulati A, Jabbour A, Ismail TF, Guha K, Khwaja J et al. (2013) Association of fibrosis with mortality and Sudden Cardiac Death in patients with nonischemic ditaled cardiomyopathy. JAMA 309: 896-908. doi:10.1001/jama.2013.1363. PubMed: 23462786. 23462786 10.1001/jama.2013.1363

[18] Pfeffer MA, Braunwald E (1990) Ventricular remodeling after myocardial infarction: experimental observations and clinical implications. Circulation 81: 1161-1172. doi:10.1161/01.CIR.81.4.1161. PubMed: 2138525. 2138525 10.1161/01.cir.81.4.1161

[19] Cohn JN, Ferrari R, Sharpe N (2000) Cardiac Remodeling – concepts and clinical implications: a consensus paper from an international forum on cardiac remodeling. J Am Coll Cardiol 35: 569-582. doi:10.1016/S0735-1097(99)00630-0. PubMed: 10716457. 10716457 10.1016/s0735-1097(99)00630-0

[20] Zornoff LAM, Paiva SAR, Duarte DR, Spadaro J (2009) Ventricular remodeling after myocardial infarction: concepts and clinical implications. Arq Bras Cardiol 92: 157-164. PubMed: 19360250. 19360249 10.1590/s0066-782x2009000200013

[21] Swynghedauw B (1999) Molecular mechanisms of myocardial remodeling. Physiol Rev 79: 215-262. PubMed: 9922372. 9922372 10.1152/physrev.1999.79.1.215

[22] Fraccarollo D, Galuppo P, Schraut S, Kneitz S, van Rooijen N et al. (2008) Immediate mineralocorticoid receptor blockade improves myocardial infarct healing by modulation of the inflammatory response. Hypertension 51: 905-914. doi:10.1161/HYPERTENSIONAHA.107.100941. PubMed: 18299485. 18299485 10.1161/HYPERTENSIONAHA.107.100941

[23] Mill JG, Milanez Mda C, de Resende MM, Gomes Mda G, Leite CM (2003) Spironolactone prevents cardiac collagen proliferation after myocardial infarction in rats. Clin Exp Pharmacol Physiol 30: 739-744. doi:10.1046/j.1440-1681.2003.03906.x. PubMed: 14516412. 14516412 10.1046/j.1440-1681.2003.03906.x

[24] Santos PP, Nogueira BF, Rafacho BP, Azevedo PS, Polegato BF et al. (2012) Aldosterone is not involved in the ventricular remodeling process induced by tobacco smoke exposure. Cell Physiol Biochem 30: 1191-1201. doi:10.1159/000343309. PubMed: 23052290. 23052290 10.1159/000343309

[25] Li X, Qi Y, Li Y, Zhang S, Guo S et al. (2013) Impact of Mineralocorticoid Receptor Antagonists on Changes in Cardiac Structure and Function of Left Ventricular Dysfunction: A Meta-analysis of Randomized Controlled Trials. Circ Heart Fail 6: 156-165. doi:10.1161/CIRCHEARTFAILURE.112.000074. PubMed: 23400891. 23400891 10.1161/CIRCHEARTFAILURE.112.000074

[26] Weir RA, Mark PB, Petrie CJ, Clements S, Steedman T et al. (2009) Left ventricular remodeling after acute myocardial infarction: does eplerenone have an effect? Am Heart J 157: 1088-1096. doi:10.1016/j.ahj.2009.04.001. PubMed: 19464421. 19464421 10.1016/j.ahj.2009.04.001

[27] Udelson JE, Feldman AM, Greenberg B, Pitt B, Mukherjee R et al. (2010) Randomized, double-blind, multicenter, placebo-controlled study evaluating the effect of aldosterone antagonism with eplerenone on ventricular remodeling in patients with mild-to-moderate heart failure and left ventricular systolic dysfunction. Circ Heart Fail 3: 347-353. doi:10.1161/CIRCHEARTFAILURE.109.906909. PubMed: 20299607. 20299607 10.1161/CIRCHEARTFAILURE.109.906909

[28] Spinale FG (2002) Matrix metalloproteinases: regulation and dysregulation in the failing heart. Circ Res 90: 520-530. doi:10.1161/01.RES.0000013290.12884.A3. PubMed: 11909815. 11909815 10.1161/01.res.0000013290.12884.a3

[29] Frangogiannis NG (2012) Matricellular proteins in cardiac adaptation and disease. Physiol Rev 92: 635-688. doi:10.1152/physrev.00008.2011. PubMed: 22535894. 22535894 10.1152/physrev.00008.2011 PMC4411042

[30] Matsui Y, Morimoto J, Uede T (2010) Role of matricellular proteins in cardiac tissue remodeling after myocardial infarction. World. J Biol Chem 1: 69-80. 10.4331/wjbc.v1.i5.69 PMC3083960 21540992

[31] Kühn B, del Monte F, Hajjar RJ, Chang YS, Lebeche D et al. (2007) Periostin induces proliferation of differentiated cardiomyocytes and promotes cardiac repair. Nat Med 13: 962-969. doi:10.1038/nm1619. PubMed: 17632525. 17632525 10.1038/nm1619

[32] Shimazaki M, Nakamura K, Kii I, Kashima T, Amizuka N et al. (2008) Periostin is essential for cardiac healing after acute myocardial infarction. J Exp Med 205: 295-303. doi:10.1084/jem.20071297. PubMed: 18208976. 18208976 10.1084/jem.20071297 PMC2271007

[33] Oka T, Xu J, Kaiser RA, Melendez J, Hambleton M et al. (2007) Genetic manipulation of periostin expression reveals a role in cardiac hypertrophy and ventricular remodeling. Circ Res 101: 313-321. doi:10.1161/CIRCRESAHA.107.149047. PubMed: 17569887. 17569887 10.1161/CIRCRESAHA.107.149047 PMC2680305

